# Rethinking the foam cosmesis for people with lower limb absence

**DOI:** 10.1177/0309364617708650

**Published:** 2017-05-18

**Authors:** Nicola Cairns, Jonathan Corney, Kevin Murray, Karena Moore-Millar, Gillian D Hatcher, Saeed Zahedi, Richard Bradbury, Joe McCarthy

**Affiliations:** 1 University of Strathclyde, Glasgow, UK; 2 Chas. A. Blatchford & Sons Ltd, Basingstoke, UK

**Keywords:** Cosmesis, prosthesis design, transfemoral, lower limb, user-centred design

## Abstract

**Background and aim:**

A recent survey of people with lower limb absence revealed that patients’ satisfaction with their foam cosmesis is lower than desired. The aim of this project was to improve the lifelike appearance, functionality and durability of the cosmesis through a user-centred design methodology.

**Technique:**

Concept development and prototyping led to a new cosmesis design which features a cut-out located at the knee, inserted with an artificial patella made of a more rigid foam. It also features a full-length zip which provides easy access for maintenance. The new cosmesis was then mechanically tested for over 1 million cycles and clinically tested by six transfemoral prosthesis users over 18 patient months.

**Discussion:**

The new design is significantly more durable than the current standard model and has an enhanced lifelike appearance. It has potential to improve users’ body image and reduce costs for healthcare providers.

**Clinical relevance:**

This study contributes to practice by offering a new cosmesis design with enhanced appearance and durability, with the potential to improve patients’ body image and reduce costs associated with cosmesis fitting and maintenance.

## Background and aim

In a UK survey of 153 people with lower limb absence, patients’ overall satisfaction with their cosmesis was lower than what the medical device industry and clinical community would desire.^1^ The aim of this project was to improve both the appearance and functionality of the foam cosmesis used as a covering for prosthetic limbs in orthopaedic applications. Based on interviews and surveys of both patients and practitioners, the following priorities for improvement were identified:

- Increased life in use;
- Improved lifelike shape;
- Improved maintenance: ease of removal from prosthesis, improved access to internal components and reduced maintenance cost.

## Technique

The research adopted a user-centred product design methodology to redesign the foam cosmesis, which places emphasis on the needs of the end user at various stages of the design process. This is a recommended methodology in the field of rehabilitation products because the end solution will have been developed with patients’ needs taken into account.^2^ In alignment with this approach, the design process was conducted as follows:

1. Creation of product specification;
2. Concept generation and development;
3. Detail design and prototyping;
4. Testing and validation;
5. Product refinement.

### Creation of product specification

First, an exploration of the properties of existing polyurethane (PU) foam cosmeses^3,4^ and interviews with a range of stakeholders (amputees, prosthetic technicians, prosthetic clinicians and cosmesis manufacturers) helped to establish the specification of materials and functional behaviours required to improve function and appearance. These are summarized as follows:

- Ease of donning and doffing without damage to the cosmesis;
- Material ability to tolerate repeated strain at knee due to seated flexing;
- Comparable rigidity and feel to the human leg;
- Comparable or reduced cost;
- Comparable or reduced fitting time;
- Comparable or reduced manufacturing time.

### Concept generation and development

Initial concepts explored the possibilities of modular foam components, which would be covered by a stocking to conceal the edges of individual sections and create the appearance of a continuous material. Then, a second approach to isolate the knee region was developed through observations of knee flexion: when the knee joint flexes, only a small area of skin is stretched, while the surface of calf and thigh areas remain essentially unchanged. Similar observations have been made by space-suit designers where Iberall’s ‘lines of non-extension’ define the potential locations of rigid elements which will not restrict body movement.^5^ It was proposed that by removing foam from *within* the Iberall boundary around the knee (i.e. the volume which experiences the most stretching during flexion of the knee joint), the strain experienced by the remaining cosmesis material would be minimized during use. In other words, the removal of the most highly stressed area of foam would prevent the initial tearing caused by wear and fatigue damage. But perhaps more importantly it would reduce the resistance to flexion to almost zero. With no foam material to deflect, the only movement to overcome would be that caused by a stocking cover.

However, a ‘knee cut-out’ will, of course, create a gap across which any covering material will stretch, resulting in an un-natural appearance. A key step in the design process was the realization that this problem was also an opportunity to enhance the lifelike appearance and behaviour of the prosthesis by incorporating an artificial patella into the knee cut-out.

Interviews with prosthetists suggested that the fitting process frequently resulted in damage to the foam. Therefore, the concept of incorporating a full leg-length zip emerged from a brainstorming session.

### Detail design and prototyping

**Figure 1.**
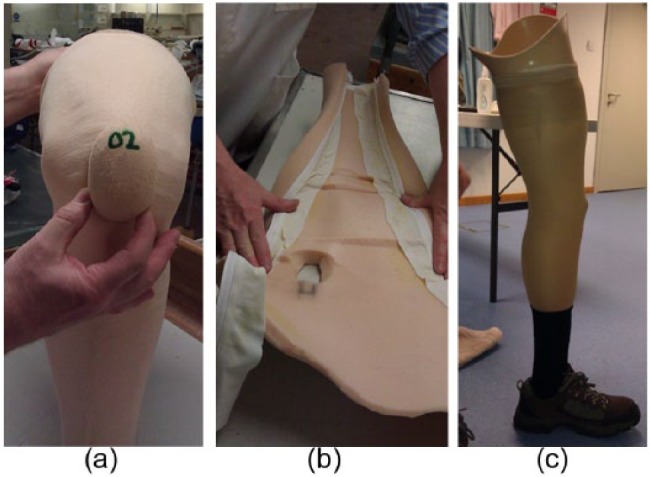
Prototyping: (a) the floating patella inserted in the knee cut-out, (b) the zip glued to the inside of the vertical length of the cosmesis and (c) the full leg-length zip adequately concealed by the stocking cover.

The feasibility of the concept was investigated through a series of trial prototypes that established an artificial patella (made of Plastazote^®^), which could be reliably located in the knee cut-out with a covering stocking over the outside and a Lycra patch internally (Figure 1).

### Final design

The final design features one cut-out located at the approximation of the anatomical patella. It also has an artificial patella, made of a more rigid foam, inserted into the cut-out. During movement, the foam cosmesis deforms while the artificial patella moves as a rigid body, reducing the level of strain and tension applied to the cosmesis foam in the knee joint area.

**Figure 2.**
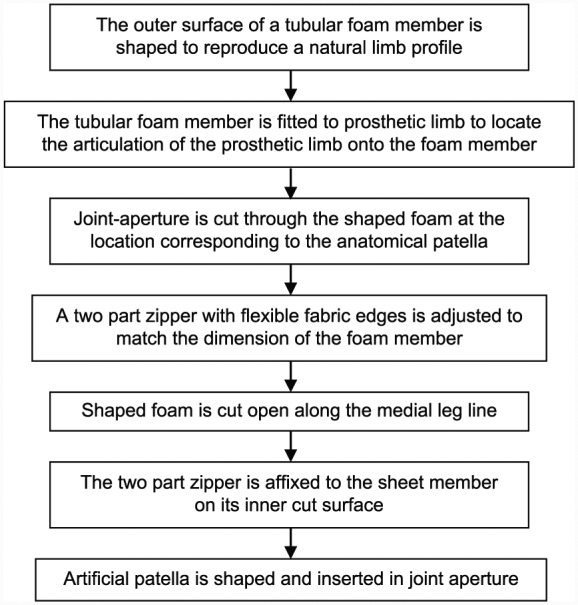
Cosmesis manufacturing process.

The design also features a full leg-length zip which provides easy access for maintenance with minimal potential for damage to the foam cosmesis. This zip is fitted to the medial inner surface of the foam cosmesis so that when fastened, the edges of the foam meet and the zip is concealed, making the cosmesis appear seamless. Prototype trials established that this feature worked well as long as the internal bore of the cosmesis was enlarged to allow easy closure (i.e. without excessively stressing the foam). The manufacturing process is outlined in Figure 2.

### Testing, validation and refinement

**Figure 3.**
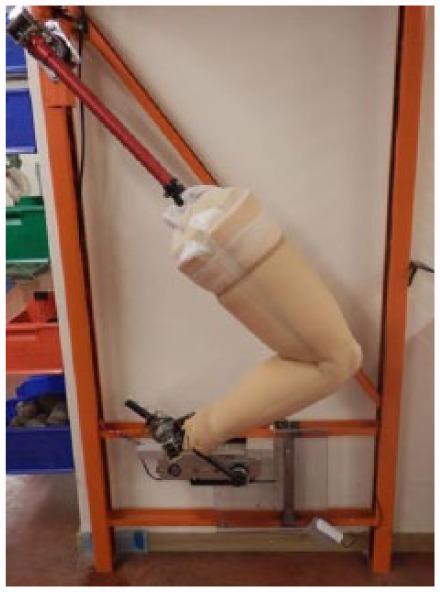
Durability test rig.

The durability of the prototype cosmesis was tested with the use of a mechanical test rig. The cosmesis was fitted to a prosthetic limb that was linked to a motor drive. The rig manipulated the prosthetic limb through a typical bicycling motion, from 15° to 130° knee flexion, before extending the knee joint to return to the original position (Figure 3). The rig was also fitted with pedometers and a magnetic counter to record the number of cycles achieved before cosmesis failure. Failure was defined as a foam rupture visible on the external surface of the cosmesis. Foam rupture was identified using 24-h video recording, focused on the knee joint area where rupture typically occurs. Four prototypes were tested to failure.

Three control cosmeses (standard, unshaped models of identical dimensions and material properties to the prototype cosmeses) were also tested using the same protocol. The control cosmeses had a mean failure rate of 352,666 cycles.

The prototype cosmesis first displayed minor material damage at approximately 100,000 cycles and had a mean failure rate of over 1 million cycles, clearly demonstrating that the knee cut-out had significant positive impact upon the cosmesis life in use.^6^

According to participant questionnaires (see below), standard cosmesis typically lasts between 7 and 12 months before rupturing at the knee joint, requiring maintenance or replacement. Based on the durability testing results, the proposed design could last around three times longer than the cosmesis design currently employed by the UK National Health Service (NHS).

The prototype cosmesis design was tested with six volunteers, who were not known to the researchers. The trials took place over a total of 18 patient months at the National Centre for Prosthetics and Orthotics, University of Strathclyde, and also at clinical sites of the project industrial partner (Chas A Blatchford Ltd). University ethical approval was granted for the trials (UEC13/58), and all volunteers provided written consent to being included in the study. Participants provided feedback through a questionnaire using a 5-point Likert scale (satisfied to dissatisfied), evaluating durability, comfort and aesthetics when sitting, kneeling and engaging in day-to-day activities. They completed this regarding their standardized cosmesis at the beginning of the trial and again regarding the new cosmesis at 4- and 10-week reviews.

After 10 weeks, the artificial patellas remained in the correct location with no evidence of wear and tear. The patellas also made kneeling more cushioned and comfortable, for example, when tying shoelaces, and did not interfere with the functionality of the prosthesis. Participants rated the new cosmesis positively for shape match to sound limb and range of knee joint movement, when compared to a standard model. The shape of the knee when flexed was rated equally when compared to a standard model.

Some minor deformation was noted on the artificial patella, as the foam material was compressed with repeated kneeling. However, one of the advantages of the new design is this component is easily replaced.

Participants agreed that the zip feature reduced the time required for prosthesis maintenance checks. They agreed that the zip was well concealed with negligible effect upon the look of the cosmesis once the stocking had been fitted.

However, a number of small rips in the foam were identified where the fabric of the zip joined the foam material, and it was reported that the zip was prone to getting stuck. As a result, a new zip component was proposed to alleviate these problems.

**Figure 4.**
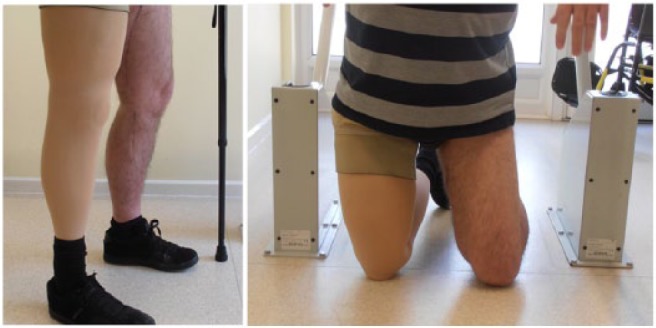
Fully assembled cosmesis undergoing clinical testing.

Alongside the needs of the user, it is also important to consider the needs of the clinical staff who prescribe and fit the cosmesis.^7^ Three professionals from a leading prosthetic manufacturer reviewed the prototype design: a clinical manager (responsible for patient satisfaction and managing budgets), a clinical specialist with expertise in patient satisfaction and comfort and a lead technician (technical expertise and responsible for cosmesis quality). Clinicians provided feedback through two questionnaires. The first was issued at the initial fitting appointment and asked them to compare the initial fitting process of the standardized cosmesis with the new cosmesis. The second was issued at the follow-up appointment and asked them to rate the durability of the new cosmesis design after a period of use. At the initial fitting, clinical staff expressed negative opinions about the prototype cosmesis because it did not reduce manufacturing time (typically 3–4 h), they did not believe the zip would improve ease of maintenance and were not convinced by the aesthetics. However, following an inspection after the 10-week trial, clinical staff provided positive feedback. The prototype cosmesis was inspected by removing it from its prosthesis and replacing (‘donning and doffing’). They found no evidence of wear and tear and agreed that the zip made it easier to don and doff the cosmesis (although there were issues with the design as noted above). They also agreed that the artificial patella improved the appearance of the cosmesis and the flexibility of the foam. The final and fully assembled cosmesis design is shown in Figure 4.

## Discussion

Testing and validation demonstrated that the new design had improved durability, lasting up to three times longer than a standard cosmesis design. The added complexity of the new design will increase manufacturing time and could mean that the initial fitting session will take slightly longer than a standard ‘tubular’ cosmesis design. However, a more durable cosmesis would reduce the frequency of clinical visits each patient requires. The zip feature of the new cosmesis was also found to improve the ease of access to the prosthesis for maintenance, providing benefits to both patients and clinicians if maintenance can be conducted in less time with less risk of damage in the process. Furthermore, the more lifelike appearance of the cosmesis could also help with psychological rehabilitation and contribute to an improved body image for prosthesis users.

The new cosmesis design may also reduce overall costs for healthcare providers, when compared to current models. A more durable design means that patients will require fewer cosmesis fittings throughout their lifetime, reducing the number of clinical visits required as well as manufacturing quantities. Manufacturing costs per unit will be higher than that of a standard cosmesis; however, the new design provides added benefits to the user at a fraction of the cost of a fully customized cosmesis. Additional information and detailed instructions for manufacture of the new cosmesis have been archived at http://dx.doi.org/10.15129/b15474d6-ffa7-4f57-b0c3-ac9f08dda7cd

## Key Points

- Low Cost;
- Better performance when kneeling;
- More realistic looking knee;
- Reduction in the effect of a new cosmesis on the swing characteristics of a limb;
- Cosmesis lasts three times longer than traditional design.

## References

[1] Cairns N Murray K Corney J et al Satisfaction with cosmesis and priorities for cosmesis design reported by lower limb amputees in the United Kingdom: instrument development and results. Prosthet Orthot Int 2014; 38: 467–473. 24327666 10.1177/0309364613512149 PMC4230545

[2] Wilkinson CR De Angeli A. Applying user centred and participatory design approaches to commercial product development. Design Stud 2014; 35: 614–631.

[3] Torres-Sánchez C Corney J. Identification of formation stages in a polymeric foam customised by sonication via electrical resistivity measurements. J Polym Res 2009; 16: 461–470.

[4] Cairns N Corney J Murray K Mechanical testing of polyurethane foams to cover lower limb prostheses. In: Proceedings of the Orthopadie + Reha-Technik 2012, Leipzig, 15–18 May 2012.

[5] Bethke KKA The second skin approach: skin strain field analysis and mechanical counter pressure prototyping for advanced spacesuit design. Cambridge, MA: Massachusetts Institute of Technology, 2005.

[6] Murray K Corney J Moore-Millar K Cairns N Extending the life and improving the appearance of cosmetic foam covers for people with trans-femoral amputations. In: ISPO 2015 World Congress, Lyon, International Society for Prosthetics and Orthotics, 2015, p. 198.

[7] Resnik L. Development and testing of new upper-limb prosthetic devices: research designs for usability testing. J Rehabil Res Dev 2011; 48: 697–706. 21938656 10.1682/jrrd.2010.03.0050

